# EFFECT OF OMEGA-3 FATTY ACID IN THE HEALING PROCESS OF COLONIC ANASTOMOSIS IN RATS

**DOI:** 10.1590/S0102-6720201500040010

**Published:** 2015

**Authors:** Tiago Jacometo Coelho de CASTILHO, Antônio Carlos Ligocki CAMPOS, Eneri Vieira de Souza Leite MELLO

**Affiliations:** 1 Postgraduate Program in Surgery, Health Sciences Sector, Paraná Federal University of Paraná, Curitiba, PR; 2 Laboratory of Morphophysiological Science, Department of Morphophysiological Science, State University of Maringá, Maringá, PR, Brazil

**Keywords:** Omega-3, Wound healing, Colonic anastomosis, Rats

## Abstract

***Background*:**

The use of long-chain polyunsaturated fatty acids has been studied in the context of healing and tissue regeneration mainly due to its anti-inflammatory, immunoregulatory and antioncogenic properties. Previous studies have demonstrated beneficial effects with the use of enteral immunonutrition containing various farmaconutrients such as L-arginine, omega-3, trace elements, but the individual action of each component in the healing of colonic anastomosis remains unclear.

***Aim*:**

To evaluate the influence of preoperative supplementation with omega-3 fatty acids on the healing of colonic anastomoses of well-nourished rats.

***Methods*:**

Forty Wistar adult male rats, weighing 234.4±22.3 g were used. The animals were divided into two groups: the control group received for seven days olive oil rich in omega-9 oil through an orogastric tube, while the study group received isocaloric and isovolumetric omega-3 emulsion at a dose of 100 mg/kg/day, also for seven days. Both groups were submitted to two colotomies followed by anastomosis, in the right and left colon, respectively. Parameters evaluated included changes in body weight, anastomotic complications and mortality, as well as maximum tensile strength by using a tensiometer and collagen densitometry at the anastomotic site.

***Results*:**

There were no differences in body weight or mortality and morbidity between groups. The value of the maximum tensile strength of the control group was 1.9±0.3 N and the study group 1.7±0.2, p=0.357. There was, however, a larger amount of type I collagen deposition in the study group (p=0.0126). The collagen maturation índex was 1.74±0.71 in the control group and 1.67±0.5 in the study group; p=0,719).

***Conclusions*:**

Preoperative supplementation of omega-3 fatty acid in rats is associated with increased collagen deposition of type I fibers in colonic anastomoses on the 5^th^ postoperative day. No differences were observed in the tensile strength or collagen maturation index.

## Abstract (pt)

***Racional*:**

O uso de ácidos graxos poliinsaturados de cadeia longa tem sido estudado no contexto de cicatrização e regeneração do tecido, principalmente devido a suas propriedades imunorreguladoras, antioncogênicas e anti-inflamatórias. Estudos anteriores demonstraram efeitos benéficos com o uso de imunonutrição enteral contendo vários farmaconutrientes (L-arginina, ômega-3, oligoelementos), mas a ação individual de cada componente na cicatrização de anastomose colônica permanece incerto.

***Objetivo*:**

Avaliar a influência da suplementação no pré-operatório com ácidos graxos ômega-3 na cicatrização de anastomoses colônicas de ratos eutróficos.

***Método*:**

Quarenta ratos Wistar, machos adultos, pesando 234,4±22,3 g foram divididos em dois grupos: grupo controle recebeu a suplementação de azeite de oliva (rico em ômega-9) por gavagem por sete dias e o grupo estudo recebeu ômega-3 em forma de emulsão isocalórica e isovolumétrica, também por gavagem, na dose de 100 mg/kg/dia por sete dias no pré-operatório. Ambos os grupos foram submetidos à duas colotomias seguidas de anastomose colônica, em cólon direito e cólon esquerdo, respectivamente. Foram avaliados a evolução do peso dos animais, morbimortalidade e realizados testes tensiométrico e de densitometria do colágeno dos corpos de prova.

***Resultados*:**

Não houve diferenças na evolução do peso e na morbimortalidade entre os grupos. O valor da força de tração máxima do grupo controle foi de 1,9±0,3N e no grupo estudo de 1,7±0,2, p=0,357. Houve, no entanto, maior quantidade de colágeno tipo I (p=0,0126) na anastomose no grupo estudo. O índice de maturação do colágeno foi cálculado para os grupos (1,74±0,71 - grupo controle; 1,67±0,5 - grupo estudo, p=0,719).

***Conclusões*:**

A suplementação pré-operatória com ácido graxo ômega-3 está associada ao aumento da deposição de colágeno do tipo I em anastomoses de cólon em ratos no 5º dia do pós-operatório, mas não exerce influência na resistência tênsil de colorrafia ou no índice de maturação do colágeno.

## INTRODUCTION

Immunonutrition is defined as the use of specific nutrients in formulas which promote through different mechanisms of action, effects on the inflammatory and imune systems^4^^,^ 10 ^,^ 14 ^,^ 22.

Initially tested in animal models, these formulas were started to be used in patients previously malnourished that were to be submitted to surgical procedures. Clear benefits were obtained on postoperative recovery, with reduced morbidity rate, length of hospital stay and duration of mechanical ventilation in intensive care units^21^. However, the use of these formulas has spread and studies began to show positive effect also in well-nourished individuals^8^^,^ 15 ^,^ 23 ^,^ 24 ^,^ 25 ^,^ 26. 

However, due to the complex mechanism of interaction between absorption processes, membranes attachment and intracellular signaling, it is known that formulas consisting of amino acids, essential fatty acids, trace elements, vitamins, nucleotides and nucleosides, were effective when using together, while the use of specific nutrients alone was associated with less clear benefits and with divergent results^5^. 

The use of long-chain polyunsaturated fatty acids have been studied in the context of healing and tissue regeneration, mainly due to its anti-inflammatory, immunoregulatory and antioncogenics properties. However, studies performed in rat models of induced colitis resulted in negative results, in which the use of omega-3 was detrimental, resulting in deterioration of colitis^16^^,^ 29. 

Its anti-inflammatory properties arise from processes such as the partial replacement of arachidonic acid in cell membranes, and thus, a reduction in the production of derivatives considered pro-inflammatory, such as prostaglandin E2. There is also a reduction in the chemotaxis of monocytes and neutrophils, modulating the inflammatory response in its earliest stages. Also notable are its immunoregulatory effects by reducing the proliferation of cytotoxic T lymphocytes in response to proinflammatory cytokines, the production of mediators such as nitric oxide and tumor necrosis factor^1^^,^ 2 ^,^ 13 ^,^ 28. 

Thus, despite the fact that omega-3 fatty acids play a clear role in the inflammatory response, the supplementation with omega-3 and its role in the overall context of colonic anastomosis healing is still unknown^4^.

The aim of this study was to evaluate the influence of preoperative supplementation with omega-3 fatty acids on the healing of colonic anastomoses in rats.

## METHODS

Forty Wistar adult male rats ( *Rattus norvegicusalbinus* , Rodentia mammalia), weighing 234.4±22.3 g obtained from the Universidade Estadual de Maringá were acclimatized individually receiving ad libitum standard rat chow prior to the surgical procedure. Seven days before surgery, the control group (GAO) received daily olive oil supplementation while the study group (GOM), received omega-3 fatty acid emulsion as an isocaloric and isovolumetric emulsions at a dose of 100 mg/kg/day through an rigid orogastric tube. 

Both groups received the fatty acids suplementation through an orogastric gavage without anesthesia and were submitted to two colotomies followed by anastomosis with interrupted 6-0 nylon in the right and left colon, respectively. On the 5^th^ postoperative day, rats were killed. Parameters evaluated included changes in body weight, anastomotic complications and mortality, as well as maximum tensile strength by using a tensiometer and collagen densitometry at the anastomotic site. Statistical analysis was performed using the Student t test, Mann-Whitney test, Fisher's exact test and Z-test to compare proportions, with p<0.05. 

## RESULTS

Anastomotic leakages occurred in four animals in each group. The mortality rate in the GAO group was 35.0% (n=7) and 10.0% (n=2) in the GOM group, p=0.1973. There were no diferences in the average weight at the beginning of the experiment, on the 7^th^, 14^th^, 21^st^ day, the day of surgery and the day of sacrifice, respectively (Table 1). 

**TABLE 1:** - Average weight±standard error (SE) per group (g)

**Days**	**GAO (n=20)**	**GOM (n=20)**	**p**				
	**Average**	**±**	**SE**	**Average**	**±**	**SE**	
D0	292,3	±	4,4	284,3	±	5,3	0,343
D7	322,3	±	5,0	313,1	±	5,6	0,208
D14	348,4	±	5,5	339,0	±	6,5	0,323
D21	369,7	±	6,2	363,4	±	6,9	0,695
DCx	349,7	±	6,4	344,0	±	7,2	0,561
DSx	321,0	±	8,8	316,1	±	3,8	0,857

D0=first day of study; DCx=day of surgery; DSx=day of sacrifice; GOM=omega-3 group; GAO=olive oil group; p=statistical signicance (Mann-Whitney test)

**FIGURE 1:**
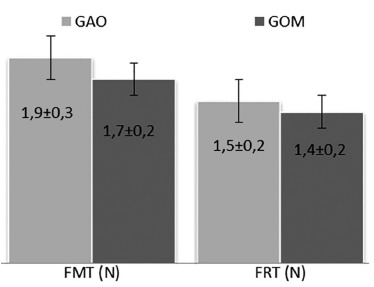
- Comparison between maximum tensile strength of the GAO and GOM groups

The value of the maximum tensile strength of the GAO group was 1.9±0.3 N and the GOM group 1.7±0.2, p=0.357 (Figure 1). The rupture force of groups GAO and GOM were also similar (1,5+0,2 vs 1,4+0,2 respectively, p=0,3572).

p=statistical significance; FMT=maximum strenght rupture force (p=0,6028); FRT=total rupture force (p=0,3572); N=newtons; GAO=olive oil group; GOM=omega-3 group (Mann-Whitney test)

**FIGURE 2:**
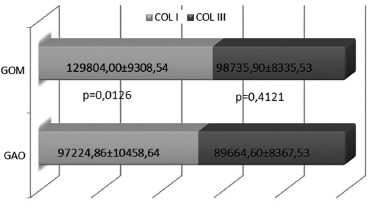
- Type I and III collagen distribuition among GOM and GAO groups

There was, however, a larger amount of type I collagen deposition (p = 0.0126) in the GOM group (Figure 2).

COL I=type I collagen;COL III=type III collagen; GAO=olive oil group; GOM=omega-3 group; SEM=standard error; p=statistical significance

The collagen maturation index (CMI) at the anastomotic site was calculated for both groups (GAO - 1.74±0.71; GOM - 1.67±0.5; p=0,719) and no difference were detected (Table 2).

**TABLE 2:** - Average±standard error for collagen maturation index for GAO and GOM groups

**Groups**	**CMI**	**p**			
	**n**	**Média**	**±**	**EP**	
GAO	13	1,74	±	0,71	0,719
GOM	18	1,67	±	0,5	

CMI=collagen maturation index; GAO=olive oil group; GOM=omega-3 group; SEM=standard error; p=statistical significance (Mann-Whitney test)

## DISCUSSION

The wound healing of the intestine after an anastomosis depends on balanced reconstitution, proliferation and differentiation processes.

The intestinal cells can be injured by surgery, inflammatory diseases, or toxic luminal substances, despite of its barrier function. The healing process starts with de surrounding epithelial cells that loses their columnar polarity, becoming a flattered cell that migrates into the denude area to restore the intestinal barrier^12^ and despite where the healing process is located, the phases of healing tends to occur in a certain sequence: inflammatory, proliferation and maturation. Each phase has its own particular duration and cell-type envolved.

 Cellular types such as neutrophils, macrophages, fibroblasts and even platelets play a fundamental role through secretion of chemoatrative factors, cell-to-cell interations and are vunerable to a vaste type of interferences, promoted mostly by the nutritional status of the host, presence or not of inflammatory process and oxidative stress. In this case the primary proinflammatory cytokines such interleukin-1 (IL-1), IL-6 and tumor necrosis factor-α (TNF- α) and also the autocrine, paracrine and endocrine events can be affect by such factors^17^. 

 All these interations of proinflammatory cytokines assist in controlling infection and prepare tissue for repair by promoting and enhacing phagocytic activity, stimulating keratinocyte migration, fibroblast chemotaxis and by regulating the remodulation of the extracellular matrix proteins^12^^,^ 17.

In this scenario, the role of immunonutrition on the colonic anastomotic healing depends on physiological performance and specific molecular interactions of the nutrients involved. Both macro and micronutrients with immuno-modulating activities have been identified, and the rationale for the use of such formulations in surgical patients is the need to reduce or mitigate the inflammatory response at specific times during surgical recovery^22^. 

Inflammation is part of the healing process.The omega-9 olive oil was chosen as a isocaloric control because of its neutral activity as inflammation is concerned. The use of the more usual soybean oil, presented in most lipid emulsions, could result in an stimulation of the inflammatory process due to its rich content of linoleic acid, a pro-inflammatory precursor.

Many studies have shown clear benefit of the use of immune- modulating enteral formulas by various mechanisms, because they enhancing the immune status in its various aspects^11^. However, the use of individual components present in these formulas did not bring such positive evidences and literature is divergent as regards to its results^4^^,^ 5. The omega-3 polyunsaturated fatty acids have been shown to play an important role in the mechanisms involving cellular signaling pathways that culminate with an anti-inflammatory effect. For example, they reduce TNF-α production by macrophages, as well as the production of pro-inflammatory cytokines and nitric oxide^1^^,^ 7 ^,^ 9 ^,^ 18. 

The immune response ability to generate an inflammatory response is eventually modified by the addition of omega-3 polyunsaturated fatty acids in the diet. The cell membrane modifies its characteristics, showing better fluidity, lipid domain areas, and a greater or lesser affinity for certain pathways which ultimately modulates especially leukocyte function. As a result, there is a competition for the same enzymatic pathways as arachidonic acid (AA), but generating less inflammatory eicosanoids. It also reduces the ability of macrophages to present antigens^1^^,^ 3 ^,^ 6 ^,^ 13 ^,^ 17 ^,^ 20 ^,^ 27 ^,^ 30. One possible consequence of the administration of omega-3 fatty acids would be the reduction of the healing process, particularly in the breaking strength, because it attenuates the inflammatory phase of the healing process. Therefore our finding that the administration of omega-3 did not reduce the tensile strength is remarcable.

The omega-3 can act also by competing with LTB4 receptors occupancy, blocking the transduction signals for synthesis of core protein G, or even in cell signaling through inhibition of phospholipase C activation induced by TNF-α, hampering or delaying the cellular signs^1^^,^ 27 ^,^ 30. 

This network involving pro and antiinflamatory factors among a nutricional status and exogenous administration of omega-3 fatty acids affects the balance of intestinal healing process in its various aspects that are still object of study by many researchers throughout the world.

## CONCLUSIONS

Preoperative supplementation with omega-3 is associated with increased collagen deposition of type I fibers in colonic anastomoses in rats on the 5^th^postoperative day. No differences were observed in the breaking strength or collagen maturation index.
